# Field and Laboratory Studies of the Susceptibility of the Green Treefrog (*Hyla cinerea)* to *Batrachochytrium dendrobatidis* Infection

**DOI:** 10.1371/journal.pone.0038473

**Published:** 2012-06-07

**Authors:** Laura A. Brannelly, Matthew W. H. Chatfield, Corinne L. Richards-Zawacki

**Affiliations:** Ecology and Evolutionary Biology Department, Tulane University, New Orleans, Louisiana, United States of America; University of Sao Paulo, Brazil

## Abstract

Amphibians worldwide are experiencing devastating declines, some of which are due to the amphibian chytrid fungus (*Batrachochytrium dendrobatidis, Bd*). Populations in the southeastern United States, however, have not been noticeably affected by the pathogen. The green treefrog (*Hyla cinerea*) is abundant and widespread in the southeastern United States, but has not been documented to harbor *Bd* infection. This study examined the susceptibility of *H. cinerea* to two strains of *Bd* in the lab and the prevalence of infection in wild populations of this species in southeastern Louisiana. Although we were able to infect *H. cinerea* with *Bd* in the lab, we did not observe any clinical signs of chytridiomycosis. Furthermore, infection by *Bd* does not appear to negatively affect body condition or growth rate of post-metamorphic individuals. We found no evidence of infection in surveys of wild *H. cinerea*. Our results suggest that *H. cinerea* is not susceptible to chytridiomycosis post-metamorphosis and probably is not an important carrier of the fungal pathogen *Bd* in the southeastern United States, although susceptibility at the larval stage remains unknown.

## Introduction

The amphibian chytrid fungus (*Batrachochytrium dendrobatidis, Bd*), and the disease it causes, chytridiomycosis, has been implicated as one of the main drivers of the global decline of amphibians [1], [2]. However, unlike in many other regions, amphibian species in the southeastern United States have not experienced noticeable die-offs due to *Bd* [3]. Despite the presence of *Bd* throughout the region, declines due to chytridiomycosis have not been reported [3]. Several of the region’s anurans, such as *Acris crepitans* and *Lithobates sevosus*, have experienced severe declines in parts of their ranges, but the role of *Bd* in those declines is uncertain [4]–[6].

The green treefrog, *Hyla cinerea*, was chosen for this study because it is one of the most abundant species across much of the southeastern United States [7], [8]. While the species has never been reported to have *Bd*, field sampling has been sparse. In searching the literature, we only found mention of 24 individuals having been tested across three studies [3], [9], [10]. If this species can harbor *Bd*, then it has the potential to be an important carrier in southeastern United States amphibian communities because of its abundance and widespread distribution. If *H. cinerea* is a carrier species we would expect *Bd* to occur on this species in the wild, and individuals to maintain an infection under laboratory conditions with no clinical signs of chytridiomycosis.

The first objective of this study was to determine whether *H. cinerea* is capable of contracting a *Bd* infection. This was accomplished by inoculating post-metamorphic individuals under laboratory conditions that are optimal for *Bd* growth. Our second objective was to determine whether wild-caught *H. cinerea* are infected with *Bd* in areas where the pathogen is known to occur. Because *Bd* is known to inhabit cool environments [11] and prevalence varies seasonally [12], [13], we sampled most heavily in the fall and spring, when environmental temperatures are suitable for *Bd* growth and *H. cinerea* are most active. If a *Bd* infection can be maintained in the lab, and if wild-caught individuals are found to harbor *Bd*, then *H. cinerea* may be an important carrier of *Bd* in the southeastern United States.

The third objective of this study was to test for differences in susceptibility and the course of infection between frogs infected with a native strain of *Bd* (from South Carolina) and those infected with a non-native strain (from Panama). There is some evidence suggesting that virulence varies among *Bd* isolates from different regions [14], [15]. Many species have declined in Panama due to chytridiomycosis [16] and thus Panamanian strains are hypothesized to be highly virulent. Since no amphibian declines have been attributed to chytridiomycosis in the southeastern United States [3] it has been hypothesized that strains from this area are less virulent.

## Materials and Methods

### Ethics Statement

This study and its methods were approved by Tulane University’s Animal Use and Care Committee (protocol No. 0391). Permission to collect *H. cinerea* was granted by the Louisiana Department of Wildlife and Fisheries (permit Nos. WL - Research – 201043 and LNHP-10-030).

### Field Data

**Figure 1:**
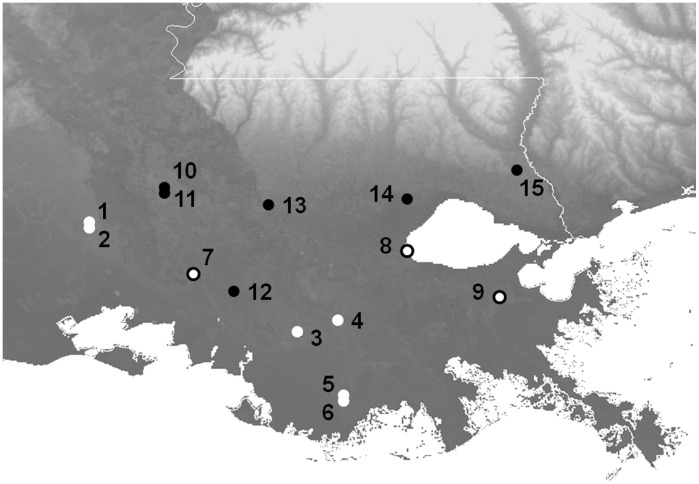
Map of field sites in southeastern Louisiana. Black circles indicate sites where only *H. cinerea* was sampled. White circles with black outlines indicate sites where *H. cinerea* and other species were sampled. White circles with no outline indicate sites where only other species (no *H. cinerea*) were sampled. Site numbers correspond to Tables 1 and S1.

**Figure 2:**
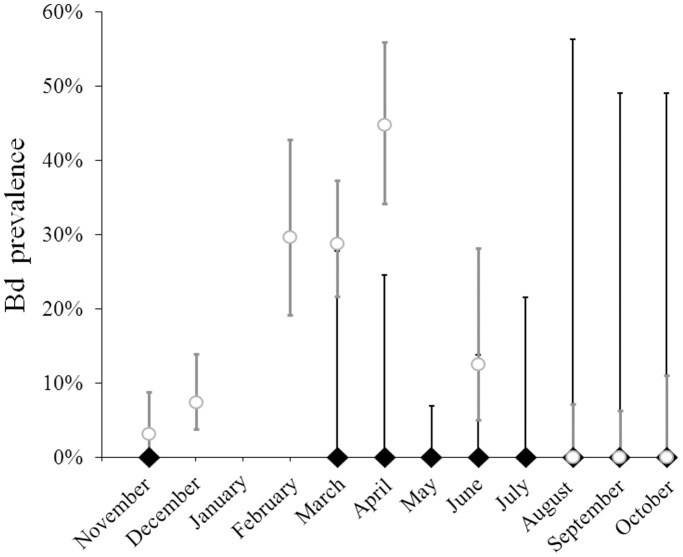
Seasonal *Bd* prevalence estimates in Southeastern Louisiana. Monthly *Bd* prevalence for all *H. cinerea* (black diamonds) and other amphibian species (open gray circles) species tested across 15 field sites. Error bars are **±**95% confidence intervals.

A total of 782 amphibians from 15 sites in southeastern Louisiana were tested for *Bd* between November 2010 and October 2011 to provide *Bd* prevalence and seasonality data (Figs. 1 and 2, Table S1). Post-metamorphic *Hyla cinerea* (juveniles and adults, n = 258) were sampled from 9 of these sites (Table 1). Animals were captured using nitrile-gloved hands, swabbed (with swab MW113, Medical Wire and Equipment Co.), and then released where they were caught. The skin surface of each frog was swabbed 40 times: five times on each of the dorsum, venter and sides, as well as the underside of each foot. Clean gloves were used to handle each animal.

**Table 1:** qPCR assay results for sites in Southeastern Louisiana where *H. cinerea* were swabbed.

Site	Site number	Latitude	Longitude	Positive/Total	Months sampled
Louisiana Immersive Technologies Enterprise Center	1	30.221	−92.043	0/24	March–September (1–5 per mo.)
National Wetlands Research Center	2	30.223	−92.045	0/14	March–May, September, October (1–6 per mo.)
Donner Canal	3	29.693	−90.966	0/5	June
Nicholls State University Farm	4	29.767	−90.784	0/11	June
Dulac Marsh	5	29.384	−90.727	0/10	July
Lower Dulac Marsh	6	29.343	−90.729	0/3	July
Sandy Cove Site 50	7	29.993	−91.526	0/2	March
Maurepas WMA	8	30.108	−90.435	0/178	November (133), May (45)
Herbert Center	9	29.89	−89.953	0/11	June, August–November (3–4 per mo.)

### Testing for *Bd*

Genomic DNA was extracted from swab samples using the Qiagen DNeasy Blood and Tissue Kit according to the instructions for animal tissue, with a final elution volume of 200 µL. Extracted DNA was then analyzed using quantitative, real-time PCR (qPCR, Applied Biosystems 7500) following Boyle et al. [17] with the following minor modifications: each sample was diluted 1∶10 with double deionized water and 0.7 µL of bovine serum albumin (BSA, Applied Biosystems) was added to each reaction well prior to amplification (following Garland et al. [18]). Positive and negative controls (extracted from swabs of known *Bd*-positive and *Bd*-negative captive *Lithobates catesbeianus,* respectively) and a series of dilution standards (provided by A. Hyatt, CSIRO, Melbourne, Australia) were included in each qPCR run. The laboratory animal samples were run in triplicate. One of the three replicates for each swab contained an internal positive control (VIC_TM_ IPC, Applied Biosystems) to ensure that PCR inhibition was not affecting our results. Samples were scored as positive if *Bd* was detected in one or more of the triplicate wells. The field animal samples were run in singlicate, where all samples contained an internal positive control.

### Laboratory Animals

Ten adult and ten juvenile *Bd*-negative *H. cinerea* were captured at Maurepas Swamp Wildlife Management Area in southeastern Louisiana in November 2010 and brought into the lab for the exposure experiment. The animals were tested and diagnosed as *Bd* negative by swabbing and qPCR analysis (see above) prior to beginning the experiment. Upon capture, adults were individually housed in 13×23×13 cm terraria. Initially, juveniles were individually housed in cylindrical terraria (11 cm diam.×14 cm tall), but by week 20 they were moved into the larger, rectangular terraria. In each enclosure, we placed 3 to 5 cm of filtered tap water. Animals were fed 1/4 in. crickets four times a week: adults were fed 16 crickets and juveniles were fed 8 crickets, until week 20 when all animals were considered adult and fed 16 crickets each. Animals were acclimated in individual terraria, inside an environmental chamber held at 20°C for at least one week prior to inoculation.

*Bd* is a biohazard and was treated as such. Materials that came in contact with fungal cultures or animals were sterilized. Terraria were sterilized once a week with a 10% bleach solution, rinsed in a water bath to remove any bleach residue, then dried completely before reuse. Nitrile gloves were changed between handling each animal in the lab and in the field to prevent cross contamination. Dry waste (e.g., gloves, swabs, paper towels) was autoclaved, and liquid waste was brought up to 10% bleach and then disposed of down the drain.

### Culturing *Bd* and Inoculation of Frogs

Animals in the laboratory study were inoculated with one of two strains of *Bd*: JEL423 or SRS810 (provided by J. Longcore, University of Maine, Bangor, ME). JEL423 is a strain isolated from a lemur leaf frog (*Phyllomedusa lemur*) in Guabal, Panama and SRS810 was isolated from a bullfrog (*Lithobates catesbeianus*) from the Savannah River Site, near Aiken, South Carolina. To prepare the inoculate, *Bd* from each strain was cultured in a 10% tryptone broth for seven days, and then transferred to 10% agar +10% tryptone plates and allowed to grow at 23°C for five to nine days. Each plate was then flooded with 5 mL of 10% Holtfreter’s solution for ten minutes to collect zoospores. The concentration of zoospores was estimated using a hemocytometer, and the inoculum was made by dilution with 10% Holtfreter’s solution. Animals were inoculated by placing them individually in 100 mL containers containing 50 mL of inoculum for 24 hours. Each animal was inoculated with *Bd* zoospores three times over the course of six days, to ensure infection. Five adult and five juvenile *H. cinerea* were inoculated with JEL423 and an additional five adults and five juveniles were inoculated with SRS810. The number of zoospores used for each set of inoculations varied depending upon how many had been produced on the agar plates during the previous week.

One week after inoculation, the first data were collected. Animals were swabbed to test for the presence of *Bd*, weighed, and measured (snout-vent length, SVL). We examined body condition (mass/SVL) as an indicator of the overall health of individuals. Data was collected every 2–3 weeks for a total of 26 weeks. Frogs were monitored daily for clinical signs of chytridiomycosis, which include the presence of red legs, loss of appetite (as indicated by number of uneaten crickets), irregular skin sloughing, or loss of righting reflex. At the end of the 26-week experimental period, all animals were euthanized using a lethal dose of MS-222.

Because terraria were changed and sterilized weekly, and amphibians shed their skin approximately every two weeks [19], individuals who returned a positive qPCR result greater than 14 d after inoculation were considered to be carrying an active infection. While a positive qPCR result cannot assure the viability of *Bd* zoospores, many of the frogs in this study tested positive for *Bd* for six months, much longer than could reasonably be attributed to carryover from the inoculation process.

## Results

### Field Study

Despite the fact that *Bd* has been detected from other amphibian species in southeastern Louisiana (Fig. 2, Table S1), all *H. cinerea* we sampled (n = 258, Table 1) tested negative for *Bd*. Many of these individuals were sampled during the same site visits as other species that tested positive, suggesting that the absence of infection in *H. cinerea* comes despite the pathogen’s presence in the local amphibian community.

### Lab Study

**Figure 3:**
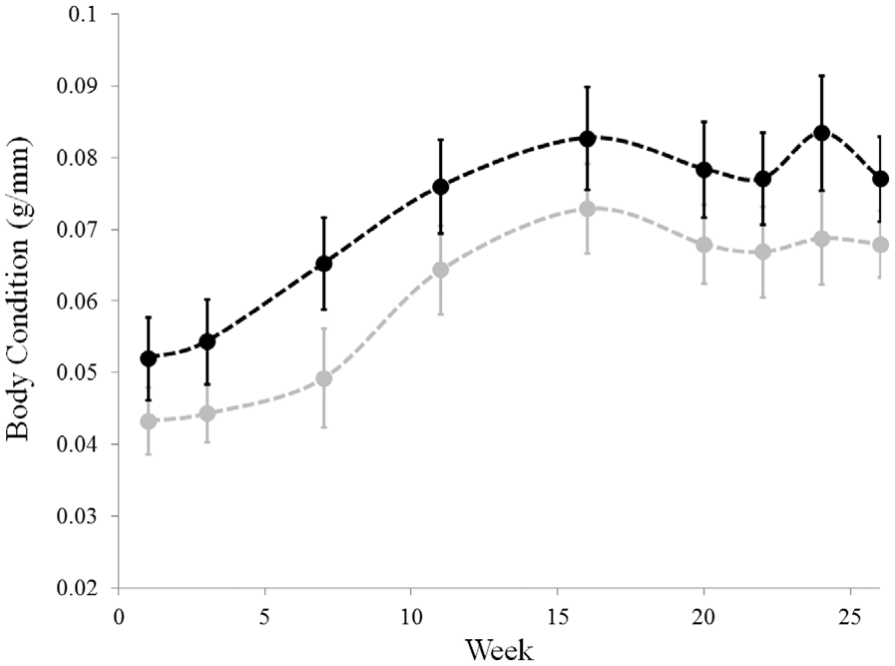
Body condition does not differ between frogs infected with different strains of *Bd*. SRS810 infected individuals: grey. JEL423 infected individuals: black. Body condition was measured mass (g) divided by SVL (mm). Week indicates the number of weeks after last inoculation. Error bars are ± one standard error of the mean.

**Figure 4:**
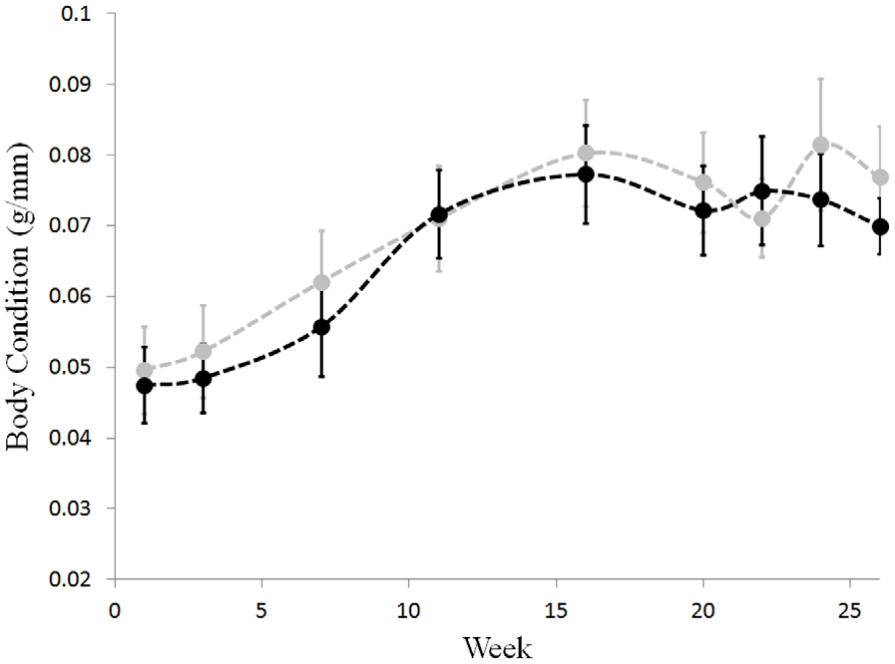
Body condition does not differ between frogs that cleared infection and those that maintained it. Infected individuals: black. Individuals that cleared their infection: grey. Body condition was measured as mass (g) divided by SVL (mm). Week indicates the number of weeks after last inoculation. Error bars are ± one standard error of the mean.

The zoospore concentrations used for inoculations varied with both strain and day. Animals inoculated with SRS810 received a dose of 2.7×10^6^ zoospores in the first inoculation, 1.6×10^6^ zoospores in the second (two days later) and 0.4×10^6^ zoospores in the third (another two days later). JEL423 animals were inoculated with 10.7×10^6^ zoospores in the first, 55.4×10^6^ zoospores in the second and 10.6×10^6^ zoospores in the third inoculation. These animals were inoculated on the same schedule as the SRS810 group. Although the inoculation doses varied between the two strains, the zoospore loads detected on the animals did not differ between the two treatment groups one week after the final inoculation (*t*-test: t_19_ = 0.73, p = 0.47). All 20 lab animals contracted a *Bd* infection; however, some individuals cleared their infection over the course of the experiment. We considered an individual to have cleared its infection if its qPCR results were consistently *Bd*-negative after week 20. Over the course of the experiment there was no difference in body condition among frogs infected with different strains of *Bd* (JEL423 and SRS810, ANCOVA: F_1,18_ = 0.036, p = 0.856, see Fig. 3). Animals that retained an infection throughout the experiment also did not differ in body condition from animals that cleared their infection during the experiment (ANOVA: F_1,18_ = 1.611, p = 0.221, see Fig. 4).

**Figure 5:**
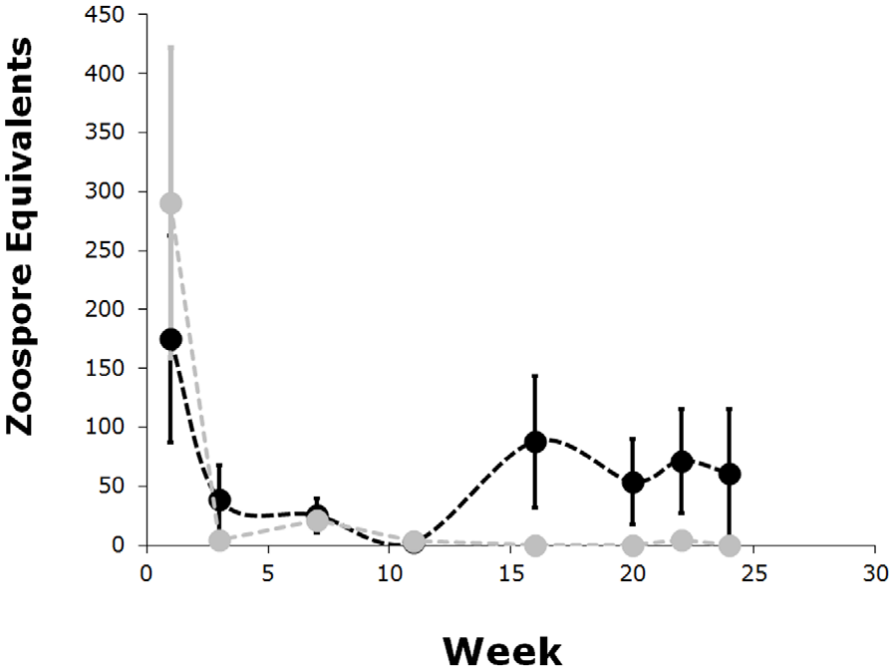
Average zoospore equivalents over time. JEL423 infected individuals: black. SRS810 infected individuals: grey. Week indicates the number of weeks after last inoculation. Error bars are ± one standard error of the mean.

The zoospore load was light for most of the animals throughout the experiment. While animals inoculated with JEL423 appeared to have higher zoospore loads near the end of the study, the pattern of variation did not differ among the two strains over the entire 26 weeks of the study (ANCOVA: F_1,18_ = 0.002, p = 0.965, see Fig. 5). Because they had very light infections, several animals tested negative for *Bd*, often times for several weeks, between positive swabs. Throughout the experiment, no animals showed clinical signs of chytridiomycosis.

## Discussion

### Inoculation

Previous inoculation studies involving *Bd* have used between 1 zoospore and 2×10^7^ zoospores [20]. Although the zoospore concentrations we used for inoculations varied among inoculation days and *Bd* strains, this did not appear to impact the infection load that resulted one week after the last inoculation. These results suggest that there may be no benefit to inoculating animals with 55×10^6^ zoospores (JEL423 at our highest dose) versus 2.6×10^6^ zoospores (SRS810 at our highest dose) in laboratory exposure experiments.

### *Bd* Infection in *Hyla cinerea*

There was no difference in infection load or disease symptoms between animals exposed to the two strains of *Bd*. Our results suggest that *H. cinerea* is able to retain an infection under laboratory conditions, but we found no evidence of any negative effects of *Bd* on infected individuals. None of the animals developed clinical signs of chytridiomycosis during the 26 week study. Because our study used an established inoculation protocol [reviewed in 20] and a *Bd* strain (JEL423) that had produced disease symptoms in other species [21], these results suggest that *H. cinerea* can carry a *Bd* infection without developing disease symptoms. Using a similar protocol and JEL423 zoospores, *Litoria caerulea* individuals succumbed to a lethal chytridiomycosis infection, as diagnosed by the presence of clinical signs and high infection loads leading to death 3–4 months after inoculation [22, and unpublished data].

All animals captured in the field tested negative for *Bd*, despite the pathogen having been detected in other amphibian species from some of the same sites and sampling sessions (Fig. 2, Table S1). Other studies that tested for the presence of *Bd* across the southeastern United States have also failed to detect infected *H. cinerea*. One such study included nine *H. cinerea* captured from four locations across the southeast, all of which tested negative [10]. In another study, 14 individuals from across four states were tested, and again, none were infected with *Bd* [3]. In both cases, infections were found in other species at the same sites. These results, in addition to those presented here, suggest that *H. cinerea* shows weak, if any, susceptibility to *Bd* in the wild.

Considering both the laboratory and field data, we conclude that *H. cinerea* is not susceptible to chytridiomycosis and *Bd* infection, at least in post-metamorphic life stages. Although individuals were able to retain a low-level infection under laboratory conditions, they did not develop disease symptoms or show any sub-clinical signs of illness. This was true for both a southeastern United States strain (SRS810) and a putatively more virulent strain associated with die-offs in Panama (JEL423) [23]. Adult and juvenile *H. cinerea* were not found to be infected with *Bd* in the field, even at sites where other species are known to be infected. If the same pattern holds for *H. cinerea* tadpoles, this suggests that the species is neither an important carrier of *Bd*, nor likely to be susceptible to *Bd*-related declines.

This study focused only on post-metamorphic life stages and therefore we cannot rule out the possibility that *H. cinerea* larvae carry *Bd* infections. Some species of anuran larvae are known to carry *Bd* infection in their keratinized mouthparts. While it is generally thought that tadpoles are not susceptible to chytridiomycosis, *Bd* infection can cause sublethal effects. For example, Garner et al. [24] found that tadpoles infected with *Bd* had a reduced mass at metamorphosis compared to uninfected tadpoles. This led them to suggest that investment of energy toward preventing *Bd* infection may overwhelm the early development process, reducing larval growth rates and even leading (directly or indirectly) to mortality at metamorphosis [24]. We do not know whether larval *H. cinerea* are susceptible to *Bd* infection; however, given that *H. cinerea* populations appear stable and abundant throughout their range, even if *Bd* infects the larval stage, it seems unlikely to be a major threat to *H. cinerea* populations.

While it is clear that amphibian species differ in their susceptibility to *Bd*, the reasons for this variation remain largely unclear. Differences in the immune system are one possible explanation for variation in susceptibility among species. This could include aspects of both the external immune system (e.g., antimicrobial bacterial colonies on the skin surface and/or antimicrobial skin peptides) and the internal immune system (e.g., lymphocytes and eosinophils) [25]–[28]. A particularly effective innate immune response in *H. cinerea* could explain the animals’ reduced susceptibility to *Bd* infections, but some other factor is then needed to explain why inoculated animals maintain an infection in the lab, while animals sampled in the field do not.

Climate and microhabitat may also influence susceptibility to *Bd*. It has been suggested that anurans that spend a large portion of their time in habitats that are ideal for *Bd* growth will be more likely to develop a *Bd* infection [29]. Post-metamorphic life stages of *H. cinerea* are largely arboreal and may not come into contact with infected water bodies as often as other species in the same habitat. Unlike in our exposure experiment, where frogs were maintained at near-optimal temperatures for *Bd* growth (20°C), wild *H. cinerea* may often be found at temperatures where the balance between immune function and zoospore growth is tipped in the frog’s favor. *Hyla cinerea* is known to bask in the sun [30], giving it one of the highest average body temperatures of arboreal frogs in the United States [30]–[32]. Basking is thought to improve digestion [33], but may also aid in eliminating chytrid infection since high temperatures are lethal to *Bd* [1], [34]–[36]. We suggest that future research should aim to tease apart the factors contributing to *Bd* resistance by focusing on species like *H. cinerea*, which do not seem to be susceptible to infection.

## Supporting Information

Table S1 *Bd* prevalence in Southeastern Louisiana.
(DOCX)
