# Supplementary material for: Field and Laboratory Studies of the Susceptibility of the Green Treefrog (Hyla cinerea) to Batrachochytrium dendrobatidis Infection
Source: PLoS One. 2012 Jun 7;7(6):e38473. doi: 10.1371/journal.pone.0038473 (PMC3369911; doi:10.1371/journal.pone.0038473)
Supplement: Table S1 — Bd prevalence in Southeastern Louisiana. (DOCX) [file pone.0038473.s001.docx]

| **Site** | **Map Symbol** | **Latitude** | **Longitude** | **Species positive/total** | **Samples positive/total** | **Months sampled** |
| --- | --- | --- | --- | --- | --- | --- |
| Sandy Cove Site 50 | 7 | 29.993 | -91.526 | 1/1 | 1/10 |  |
| *Acris crepitans* |  |  |  |  | 1/10 | March |
| Maurepas WMA | 8 | 30.108 | -90.435 | 2/2 | 4/16 |  |
| *Acris crepitans* |  |  |  |  | 3/15 | March |
| *Siren intermedia* |  |  |  |  | 1/1 | January |
| Herbert Center | 9 | 29.890 | -89.953 | 3/7 | 70/525 |  |
| *Acris crepitans* |  |  |  |  | 67/451 | February - April, June, August - December (3 - 66 per mo.) |
| *Bufo nebulifer* |  |  |  |  | 0/25 | April, June, August, October, November (1 - 5 per mo.) |
| *Gastrophryne carolinensis* |  |  |  |  | 0/8 | April, August - October (1 - 3 per mo.) |
| *Pseudacris foquettei* |  |  |  |  | 3/25 | February, June, August - December (1 - 16 per mo.) |
| *Rana clamitans* |  |  |  |  | 2/6 | February - April, November (1 - 3 per mo.) |
| *Notophthalmus viridescens* |  |  |  |  | 0/9 | March, November, December (2 - 4 per mo.) |
| *Pseudacris crucifer* |  |  |  |  | 0/1 | October |
| Bayou Manual Road | 10 | 30.408 | -91.673 | 0/2 | 0/7 |  |
| *Acris crepitans* |  |  |  |  | 0/6 | March |
| *Pseudacris crucifer* |  |  |  |  | 0/1 | March |
| ARMI Site 40 | 11 | 30.453 | -91.665 | 0/2 | 0/3 |  |
| *Acris crepitans* |  |  |  |  | 0/2 | March |
| *Rana clamitans* |  |  |  |  | 0/1 | March |
| Belle River Site 65 | 12 | 29.908 | -91.306 | 1/1 | 7/10 |  |
| *Acris crepitans* |  |  |  |  | 7/10 | March |
| Baton Rouge | 13 | 30.364 | -91.121 | 1/3 | 1/22 |  |
| *Acris crepitans* |  |  |  |  | 0/8 | March |
| *Rana clamitans* |  |  |  |  | 0/1 | March |
| *Acris gryllus* |  |  |  |  | 1/12 | March |
| *Pseudacris crucifer* |  |  |  |  | 0/1 | March |
| Joyce WMA | 14 | 30.397 | -90.429 | 0/1 | 0/1 |  |
| *Siren intermedia* |  |  |  |  | 0/1 | January |
| Talisheek Creek | 15 | 30.539 | -89.875 | 1/1 | 1/2 |  |
| *Necturus beyeri* |  |  |  |  | 1/2 | March, April |
